# Macrophagic control of the response to uropathogenic *E. coli* infection by regulation of iron retention in an IL‐6‐dependent manner

**DOI:** 10.1002/iid3.123

**Published:** 2016-08-28

**Authors:** Nana Owusu‐Boaitey, Kyle A. Bauckman, Tingxuan Zhang, Indira U. Mysorekar

**Affiliations:** ^1^ Department of Obstetrics and GynecologyWashington University School of MedicineSt. LouisMissouri; ^2^ Department of Pathology and ImmunologyWashington University School of MedicineSt. LouisMissouri

**Keywords:** Ferritin, lipocalin 2, urinary tract infection

## Abstract

**Introduction:**

Uropathogenic *Escherichia coli* (UPEC), the causative agent of over 85% of urinary tract infections (UTIs), elaborate a number of siderophores to chelate iron from the host. On the other hand, the host immune imperative is to limit the availability of iron to the bacteria. Little is known regarding the mechanisms underlying this host‐iron‐UPEC interaction. Our objective was to determine whether macrophages, in response to UPEC infection, retain extracellular siderophore‐bound and free iron, thus limiting the ability of UPEC to access iron.

**Methods:**

Quantitative PCR, immunoblotting analysis, and gene expression analysis of wild type and IL‐6‐deficient macrophages was performed.

**Results:**

We found that (1) macrophages upon UPEC infection increased expression of lipocalin 2, a siderophore‐binding molecule, of *Dmt1*, a molecule that facilitates macrophage uptake of free iron, and of the intracellular iron cargo molecule ferritin, and decreased expression of the iron exporter ferroportin; (2) bladder macrophages regulate expression of genes involved in iron retention upon UPEC infection; (3) IL‐6, a cytokine known to play an important role in regulating host iron homeostasis as well as host defense to UPEC, regulates this process, in part by promoting production of lipocalin 2; and finally, (4) inhibition of IL‐6 signaling genetically and by neutralizing antibodies against the IL‐6 receptor, promoted intra‐macrophagic UPEC growth in the presence of excess iron.

**Conclusions:**

Together, our study suggests that macrophages retain siderophore‐bound and free iron in response to UPEC and IL‐6 signaling is necessary for macrophages to limit the growth of UPEC in the presence of excess iron. IL‐6 signaling and iron regulation is one mechanism by which macrophages may mediate UPEC clearance.

## Introduction

Urinary tract infections (UTIs) are one of the most common infections worldwide and are primarily caused by uropathogenic *Escherichia coli* (UPEC) 1. Given the rising incidence of antibiotic resistance among uropathogens, there is an increasing need to better understand the host response to UPEC and to develop ways to harness the host innate immune response that clears infection. Macrophages are one such host defense entity. These innate immune cells are not only critical in response to pathogens, but also for maintaining host homeostasis 2.

Macrophages provide host cells with access to iron 2, a metal crucial for host redox reactions and for microbial survival 3, 4, 5, 6. In the absence of infection, macrophages contribute to tissue homeostasis by releasing iron 2. Macrophages take up extracellular iron bound to the cargo molecule transferrin, remove the iron from transferrin, and release this free iron into the extracellular space through the exporter ferroportin 2, 7, 8. Iron regulatory proteins (IRPs), which regulate ferroportin, transferrin receptor (TFR1), and divalent metal transporter 1 (DMT1) expression, offer another layer of control over iron availability 9, 10, 11.

However, upon bacterial infection, macrophages retain iron intracellularly by taking up free iron via DMT1 and storing this free iron in a ferritin‐bound state. Furthermore, macrophages limit the release of free iron by increasing expression of hepcidin, a negative regulator of ferroportin, and by decreasing ferroportin and TFR1 expression 2, 6, 12. In addition, macrophages also produce lipocalin 2, which binds to extracellular bacterial siderophores and is taken up by macrophages thereby limiting extracellular iron available for bacterial growth 12, 13, 14. While hepcidin and lipocalin 2 have been shown to be produced during the pathogenesis of upper UTIs 15, 16, UPEC have been shown to exploit host iron during bladder infection (cystitis) through elaborating lipocalin‐2‐resistant siderophores such as salmochelin 5, 15, 17 and accessing iron within bladder epithelial cells 18, 19. However, whether and how macrophages are involved and responsive to UPEC remain unknown. UPEC infection induces macrophages and bladder epithelial cells to produce, as a first response, interleukin 6 (IL‐6) 20, 21, a cytokine that promotes iron retention through inducing hepcidin production by liver cells 22, 23, 24. However, the role of IL‐6 signaling in macrophage iron regulation upon UPEC infection remains unknown.

We reasoned that modulation of iron availability could alter the macrophagic capacity to limit UPEC survival. We sought to test the model that macrophages mediate the inflammatory IL‐6 response to UPEC infection, in part, by producing lipocalin 2, thereby limiting iron availability to the uropathogens. We also sought to investigate the macrophage iron retention response under normal and high iron conditions, as macrophages are exposed to different iron levels over the course of an in vivo infection 4. We used primary peritoneal macrophages (pMacs) as a model to elucidate macrophage‐iron‐UPEC interactions. pMacs model monocyte‐derived recruited macrophages 25, 26 and have been previously used to investigate host regulation of iron 27, 28, 29. Here, we show that pMacs retain free iron in response to UPEC by decreasing ferroportin expression, increasing hepcidin production, and increasing *Dmt1* expression. pMacs also take up extracellular siderophore‐bound iron through production of lipocalin 2. We provide further evidence that, in response to UPEC, IL‐6 signaling inhibits iron retention and promotes lipocalin 2 production by pMacs pre‐exposed to high iron conditions. Inhibition of IL‐6 signaling promotes UPEC survival within pMacs pre‐exposed to high iron conditions, suggesting that macrophage IL‐6 signaling is necessary to limit the ability of UPEC to access iron. Finally, we show that bladder tissue macrophages isolated from mice infected with UPEC upregulate expression of genes involved in iron retention upon UPEC infection. Together, our findings suggest macrophage IL‐6 signaling and regulation of iron may limit UPEC growth.

## Results

### pMacs retain iron and limit UPEC survival

**Figure 1:**
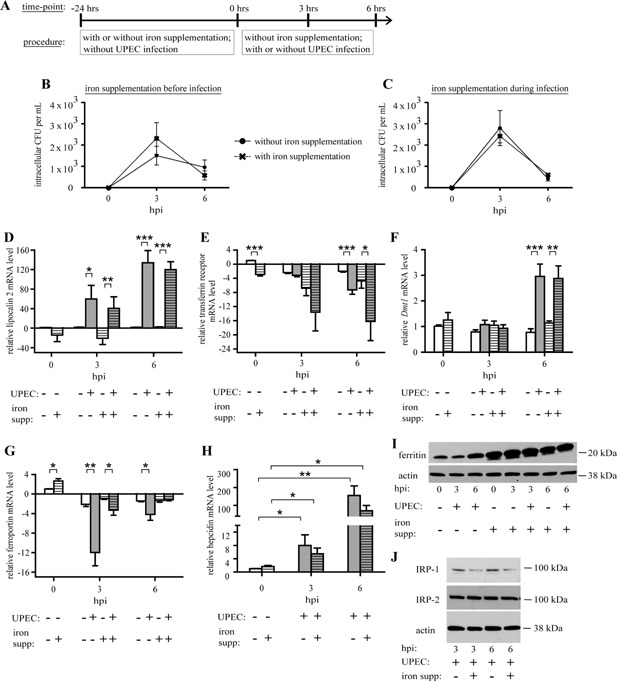
pMacs retain iron in response to UPEC, and limit UPEC utilization of iron for UPEC survival. (A) Summary of procedure for infecting pMacs with UPEC. Peritoneal macrophages (“pMacs”) were pre‐treated for 24 h with or without ferric ammonium citrate (FAC) as a source of iron for iron supplementation. The pMacs were infected with UTI89 at an MOI of 0.1 for 3 or 6 h. Intracellular CFU analysis in pMacs uninfected or infected with UTI89 at an MOI of 0.1 for 3 or 6 h in the presence of iron supplementation before infection (B) or during infection (C). qPCR analysis on mRNA expression of for lipocalin 2 (D), TFR1 (E), *Dmt1 (*F), ferroportin (G), and hepcidin (H). Western Blots for ferritin and actin (I), IRP1, IRP2, and actin (J) in pMacs (three biological replicates; qPCR values relative to uninfected pMacs without iron supplementation; compared by ANOVA; bars represent ± SEM, *P < 0.05, **P < 0.01, ***P < 0.001).

UPEC exploits host iron for its survival 5, 15, 17, including by chelating iron from within bladder epithelial cells 18, 19. We recently showed that high iron levels promote UPEC growth within bladder epithelial cells 19. UPEC can also persist within macrophages 30, 31. It remains unclear, however, whether UPEC exploits macrophages as a source of iron under high iron conditions and homeostatic iron conditions that mimic in vivo infection 4. We therefore sought to investigate the macrophage response to UPEC in the context of homeostatic levels of iron (“control”) or following iron supplementation (“iron supplemented”) resulting from the addition of ferric ammonium citrate (FAC) as a source of iron at a concentration consistent with previous publications 7, 32. We infected control and iron‐supplemented peritoneal macrophages (pMacs) with UPEC for 3 and 6 h (see schematic in Fig. 1A) and measured intracellular and extracellular bacterial CFUs. Interestingly, there was no significant difference in intracellular UPEC load within control pMacs in comparison to iron‐supplemented pMacs (Fig. 1B), nor was there a significant difference in extracellular UPEC load (data not shown). In addition, UPEC had no significant effect on pMac survival at these time‐points (data not shown). We have previously shown that iron alone promotes UPEC growth in minimal media and in bladder epithelial cells 19; thus, the UPEC growth restriction observed in macrophages is attributable to macrophage function.

We then sought to examine whether pMacs limited the ability of UPEC to exploit extra‐macrophagic free iron. To investigate this, we infected pMacs in the presence or absence of iron supplementation during the infection course. There were once again no notable differences in intracellular UPEC load within control pMacs in comparison to iron‐supplemented pMacs (Fig. 1C), nor was there a significant difference in extracellular UPEC load (data not shown). Together, this indicates that pMacs successfully prevent UPEC from exploiting free iron for their survival.

We next sought to investigate the mechanisms by which the pMacs limited the ability of UPEC to access iron. To determine this, we supplemented pMacs with iron followed by infection with UPEC for 3 and 6 h and assayed for iron retention marker expression upon each treatment. We found that iron supplementation alone did not significantly affect expression of lipocalin 2 (Fig. 1D), indicating that iron supplementation alone does not cause the pMacs to increase uptake of extracellular siderophore‐bound iron. However, upon UPEC infection, both control and iron‐supplemented pMacs increased expression of lipocalin 2 (Fig. 1D), suggesting that the infected pMacs increased uptake of extracellular siderophore‐bound iron.

On the other hand, we found that iron supplementation alone reduced expression of TFR1 and UPEC infection induced a further decrease in expression (Fig. 1E), suggesting that the pMacs may normally limit the release of free iron from its transferrin‐bound and infected pMacs further limit this release. Next, we examined *Dmt1* expression and found that iron supplementation had no significant effect while infection increased *Dmt1* expression (Fig. 1F), implying that infected pMacs took up more free iron via DMT1 in response to infection. Finally, iron supplementation alone increased expression of ferroportin (Fig. 1G), but not hepcidin (Fig. 1H), a negative regulator of ferroportin. Both control and iron‐supplemented pMacs retained free iron and limited its release in response to UPEC, as reflected in decreased ferroportin expression (Fig. 1G), increased hepcidin expression (Fig. 1H). To confirm that these expression patterns were not specific to pMacs, we repeated our experiments in bone marrow derived macrophages and observed similar patterns (data not shown). To demonstrate that the decrease in transferrin‐bound iron uptake did not suggest any defect in the pMac ability to store intracellular iron, we examined production of the iron cargo molecule ferritin. Both iron supplementation and UPEC infection increased ferritin production (Fig. 1I), suggesting that the iron‐supplemented pMacs were retaining more free iron in a ferritin‐bound state in response to UPEC. Consistent with this finding, we found that iron supplementation led to reduction in IRP1 expression, which is known to regulate ferritin production (Fig. 1J). Taken together, our data show that pMacs are adept at limiting the ability of UPEC to access host iron stores, even in the presence of excess iron.

### pMacs activate IL‐6 production to regulate iron stores in response to UPEC

**Figure 2:**
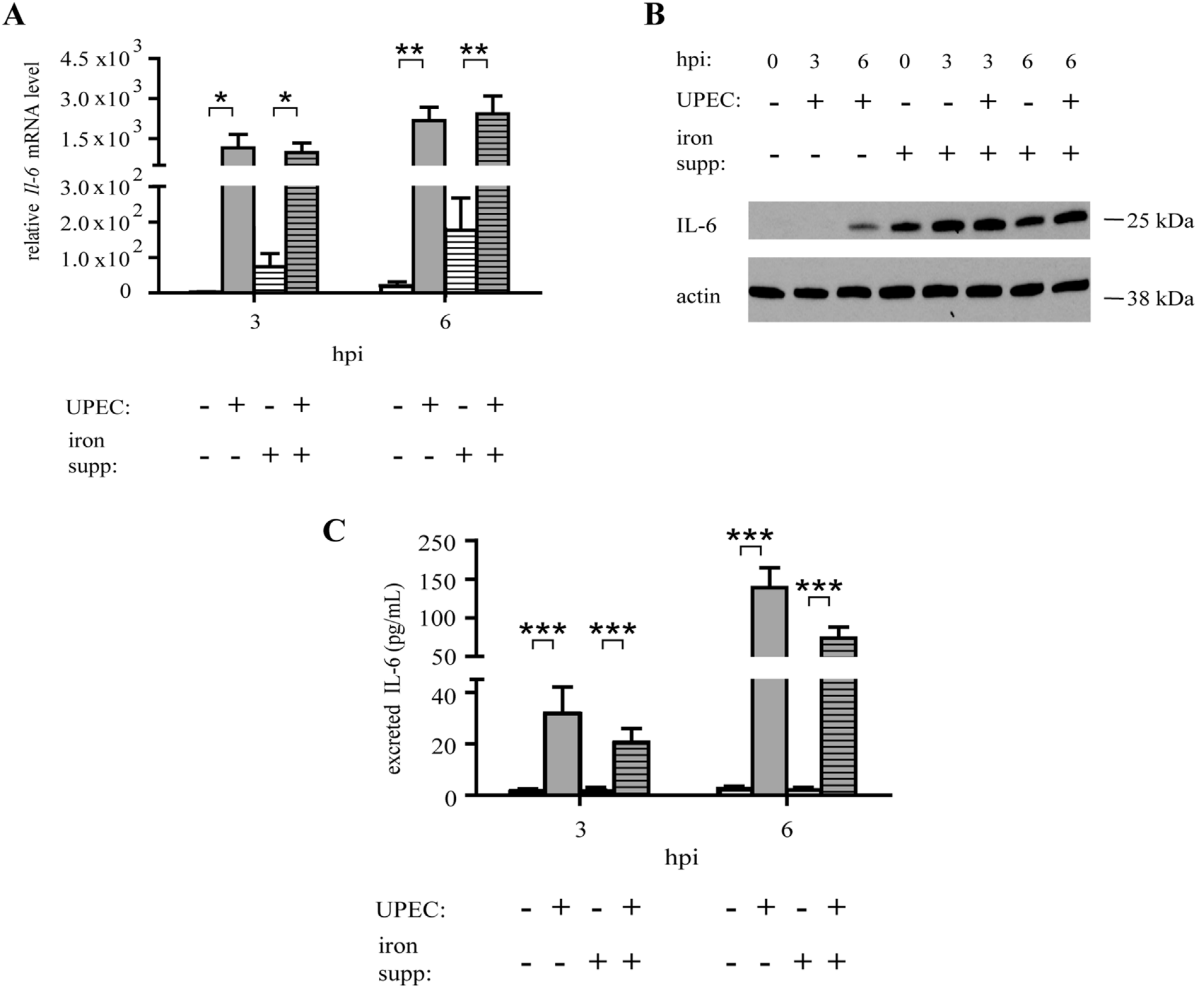
Iron and UPEC together activate induce IL‐6 production in pMacs. qPCR for *Il‐6* relative to uninfected pMacs without iron supplementation (A), Western Blot for IL‐6, ferritin, and actin, (B), and an ELISA for IL‐6 (C) in pMACs infected with UPEC after iron supplementation (three biological replicates; qPCR values relative to uninfected pMacs without iron supplementation; compared by ANOVA; bars represent ± SEM, *P < 0.05, **P < 0.01, ***P < 0.001).

Host iron homeostasis is known to be regulated by the pro‐inflammatory cytokine IL‐6, which induces hepcidin production by liver cells 23, thereby promoting iron retention. We reasoned that pMacs may use IL‐6 signaling in response to UPEC to regulate iron availability, since macrophages and bladder epithelial cells produce IL‐6 as a first response to UPEC infection 20, 21. We therefore infected iron‐supplemented and control pMacs with UPEC and determined IL‐6 production. We found, as expected, that UPEC induced *Il‐6* mRNA expression (Fig. 2A), IL‐6 protein production (Fig. 2B), and IL‐6 protein export (Fig. 2C). Interestingly, we found that iron supplementation was sufficient to activate IL‐6 production even in the absence of a bacterial stimulus (Fig. 2B), consistent with previously published data 33. However, iron supplementation did not augment IL‐6 export (Fig. 2C). Taken together, these data suggests that iron promotes the IL‐6 production pathway in response to UPEC.

### IL‐6 signaling is required for promoting iron retention in response to UPEC

**Figure 3:**
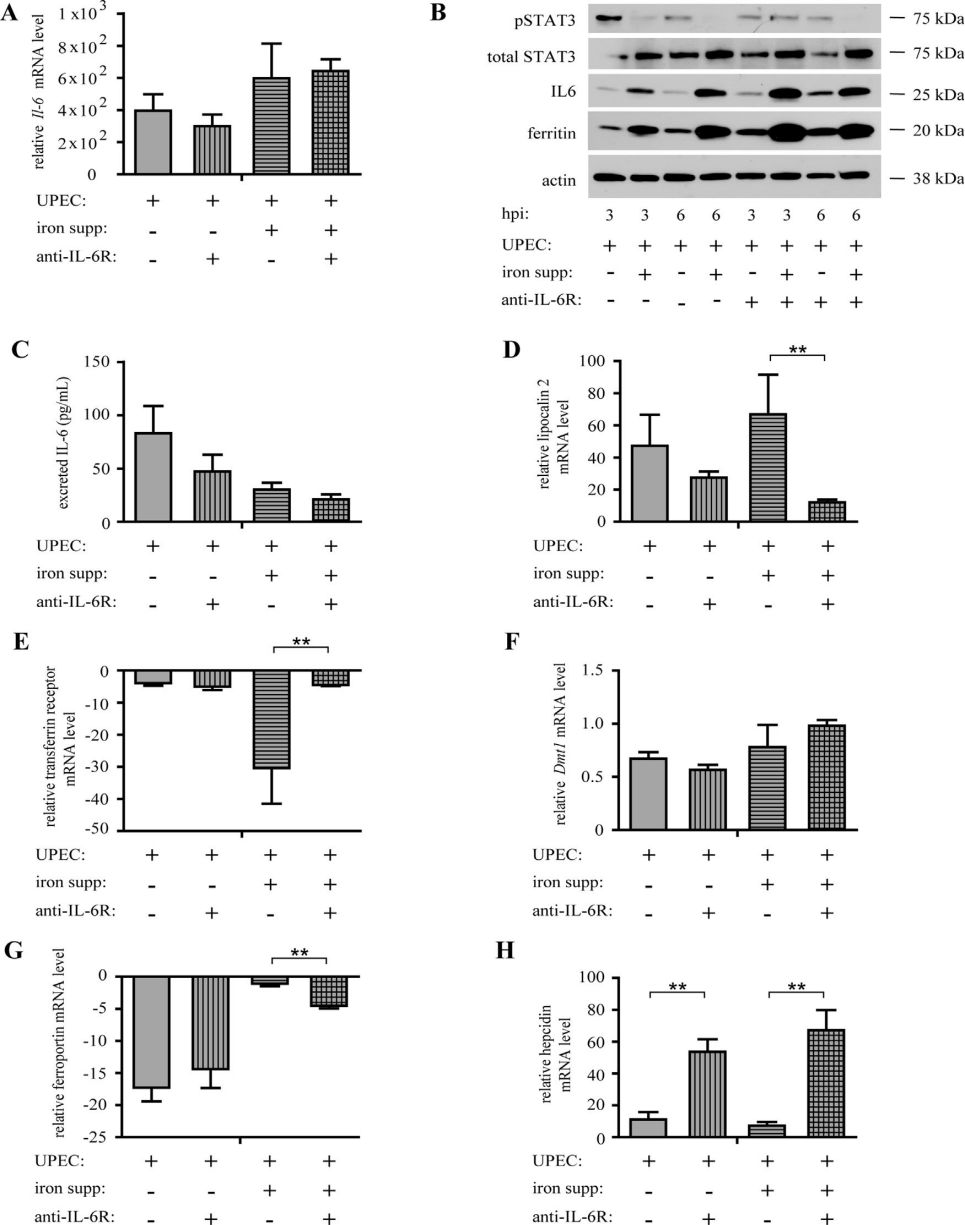
IL‐6 signaling inhibits iron retention and promotes lipocalin 2 production in UPEC infected pMacs. (A) qPCR for *Il‐6* at 3 h postinfection (hpi) in pMacs pre‐treated with or without anti‐IL‐6Rα, supplemented with iron and infected with UPEC . (B) Western Blot for IL‐6, p‐STAT3, STAT3, ferritin, and actin, (C) ELISA for IL‐6 at 3 hpi, and (D) qPCR for lipocalin 2, (E) TFR1, (F) *Dmt1*, (G) ferroportin, and (H) hepcidin at 3 pi for pMacs pre‐treated with anti‐IL‐6Rα and iron, followed by infection with UPEC (three biological replicates; qPCR values relative to uninfected pMacs without iron supplementation; compared by ANOVA; bars represent ± SEM, *P < 0.05, **P < 0.01, ***P < 0.001).

Next, we sought to determine if IL‐6 signaling plays a key role in regulating pMac iron retention in response to UPEC. To determine this, we infected control and iron‐supplemented pMacs with UPEC for 3 and 6 h. During both iron supplementation and infection, we treated the pMacs with either a control antibody or a monoclonal antibody against the IL‐6 receptor‐α subunit 34, 35, to inhibit IL‐6 signaling. We found that anti‐IL‐6Rα treatment did not significantly affect *Il‐6* mRNA expression in the context of UPEC infection (Fig. 3A). However, inhibition of IL‐6 signaling led to IL‐6 protein accumulation (Fig. 3B) and a corresponding decrease in IL‐6 protein export (Fig. 3C). We found that anti‐IL‐6Rα treatment did not significantly affect total STAT3 levels but decreased p‐STAT3 production (Fig. 3B). However, iron supplementation not only decreased p‐STAT3 production but also increased total STAT3 levels (Fig. 3B), suggesting that iron supplementation inhibited UPEC‐induced IL‐6 signaling.

Next, we determined whether inhibition of IL‐6 signaling altered pMac iron retention. We found that inhibiting IL‐6 signaling limited UPEC‐induced expression of lipocalin 2 in iron‐supplemented pMacs, but not control pMacs (Fig. 3D), suggesting that IL‐6 signaling promotes uptake of extracellular siderophore‐bound iron in iron‐supplemented pMacs only. Furthermore, anti‐IL6Rα treatment increased ferritin production (Fig. 3B), increased TFR1 expression (Fig. 3E), decreased ferroportin expression (Fig. 3G), and increased hepcidin expression (Fig. 3H) in infected iron‐supplemented pMacs. On the other hand, in infected control pMacs, anti‐IL‐6Rα treatment did not significantly affect TFR1 expression (Fig. 3E) or ferroportin expression (Fig. 3G), suggesting that IL‐6 signaling may be limiting iron retention. This inhibition of iron retention is not due to reduction in free iron uptake via DMT1, since inhibiting IL‐6 signaling did not significantly affect expression of *Dmt1* in either infected control pMacs or infected iron‐supplemented pMacs (Fig. 3F). Together, our data indicate that, in UPEC‐infected pMacs exposed to high iron conditions, IL‐6 signaling promotes uptake of extracellular siderophore‐bound iron, limits release of extracellular free iron from transferrin, and inhibits iron retention.

### pMacs require IL‐6 signaling to prevent UPEC from exploiting excess iron

**Figure 4:**
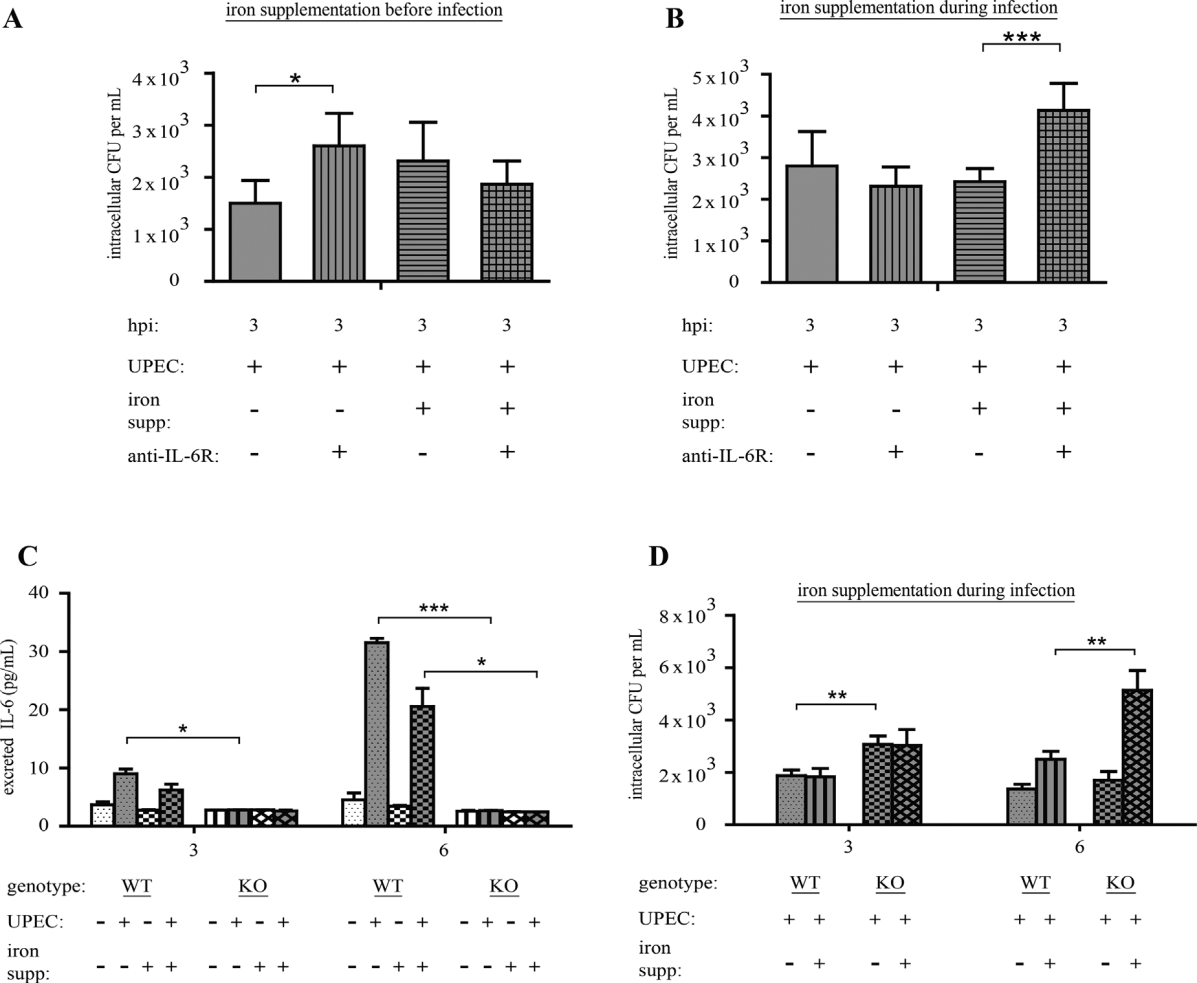
Iron‐supplemented pMacs use IL‐6 signaling to limit UPEC utilization of iron. Intracellular CFUs at 3 hpi in pMacs treated with anti‐IL‐6Rα and supplemented with iron before infection (A) or during infection (B). (C) ELISA for IL‐6 release by IL‐6^−/−^ (WT) or IL‐6^−/−^ (KO) pMacs supplemented with iron before infection (D) Intracellular CFUs in WT and KO pMacs supplemented with iron during infection (A and B: three biological replicates; C and D: two biological replicates; compared by ANOVA; bars represent ± SEM, *P < 0.05, **P < 0.01, ***P < .001).

We next sought to determine if pMacs required IL‐6 signaling in order to limit the ability of UPEC to exploit iron. To investigate this, we infected control and iron supplemented pMacs with UPEC for 3 and 6 h; during both iron supplementation and infection, we treated the pMacs with either a control antibody or anti‐IL‐6Rα. We observed that inhibiting IL‐6 signaling increased the bacterial load in control pMacs, but not iron‐supplemented pMacs (Fig. 4A). We then determined whether IL‐6 signaling was required for pMacs to limit the ability of UPEC to exploit extra‐macrophagic free iron by treating pMacs in the presence or absence of iron supplementation during the infection course, with a control antibody or anti‐IL‐6Rα added during infection. We found that inhibition of IL‐6 signaling significantly increased UPEC load only within the iron‐supplemented pMacs (Fig. 4B).

To determine if this would be the case in a background of genetic loss of function of IL‐6, we isolated pMacs from IL‐6 knockout mice (IL‐6^−/−^). We found, as expected, that WT (IL‐6^+/+^) pMacs, but not IL6^−/−^ pMacs, released IL‐6 in response to UPEC infection (Fig. 4C). However, at early as 3 h post‐infection (hpi), we observed increased bacterial loads in IL6^−/−^ pMacs (Fig. 4D). Furthermore, we determined that iron supplementation during the infection course also increased the bacterial load in IL6^−/−^ pMacs, although there was no significant difference in the absence of iron supplementation (Fig. 4D), consistent with the results we observed with anti‐IL6Rα treatment. Thus, together our data show that IL‐6 signaling limits the ability of UPEC to survive in pMacs under homeostatic iron levels as well as under conditions of high extra‐macrophagic iron.

### Bladder macrophages upregulate markers of iron retention during a UTI

**Figure 5:**
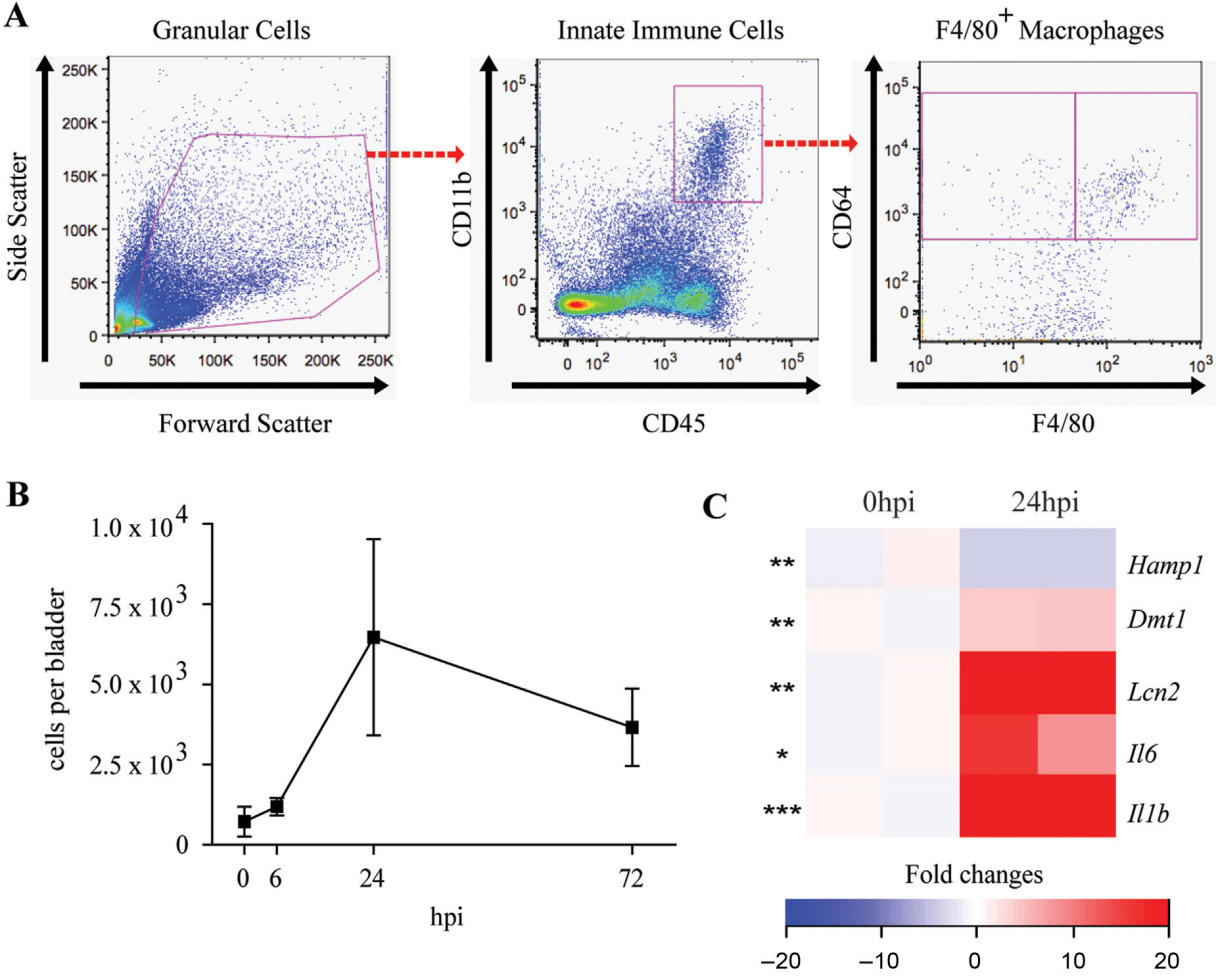
Bladder macrophages express markers of iron retention during a UTI. (A) Gating strategy innate immune cells (CD45^+^CD11b^+^) and macrophages (CD45^+^CD11b^+^CD64^+^F4/80^+^). (B) Flow cytometric analysis of the number of macrophages from uninfected mouse bladders, and UTI89‐infected mouse bladders at 6, 24, or 72 h post‐infection. (C) Microarray analysis for hepcidin (*Hamp1*), *Dmt1*, lipocalin 2 (*Lcn2*), *Il6*, and *Il1b* for macrophages from uninfected (0 hpi) and UTI89‐infected mouse bladders. The analysis depicts fold change in gene expression relative to uninfected 0 hpi samples (B and C: two biological replicates, five mouse bladders pooled per group per experiment; compared by ANOVA; bars represent ± SEM, *P < 0.05, **P < 0.01, ***P < 0.001; C: cutoff at fold change >2 between 0 hpi and 24 hpi macrophage samples).

We next investigated whether the macrophage iron retention response occurs in vivo during a UTI. This is of particular importance since inflammatory macrophages are recruited to the infected bladder mucosa upon UPEC infection 36, 37. In order to characterize the bladder macrophage compartment before and during UPEC infection, we isolated bladders from PBS‐treated control mice and mice infected with UPEC, and analyzed the bladder macrophages by flow cytometry. Bladders were isolated 6 or 24 h post‐infection (hpi) during the acute infection phase, or 72 hpi during the tissue repair phase. Macrophages were defined as granular cells positive for the hematopoietic cell marker CD45, the phagocytic receptor CD64 37, 38, the integrin CD11b 37, 39, and the mature macrophage marker F4/80 36, 37, 38, 39, 40 (Fig. 5A). Though a relatively small number of bladder macrophages were present before infection, the number of macrophages increased over the course of infection (Fig. 5B), consistent with previously published data 41.

Next, to examine whether UPEC infection regulates iron retention by macrophages, we performed microarray analysis on isolated bladder macrophages. We found that UPEC infection led to upregulation of *Dmt1* and lipocalin 2 (*Lcn2*), markers involved in iron retention (Fig. 5C). UPEC infection did not significantly affect hepcidin (*Hamp1*) expression in these macrophages (Fig. 5C). However, bladder macrophages exhibited increased expression of pro‐inflammatory cytokines that can promote iron retention, as evidenced by increased expression of IL‐6 and IL‐1β upon infection (Fig. 5C). Together, these results indicate that bladder macrophages upregulate markers of iron retention in response to UPEC infection in vivo; thereby limiting iron availability for UPEC to exploit.

## Discussion

**Figure 6:**
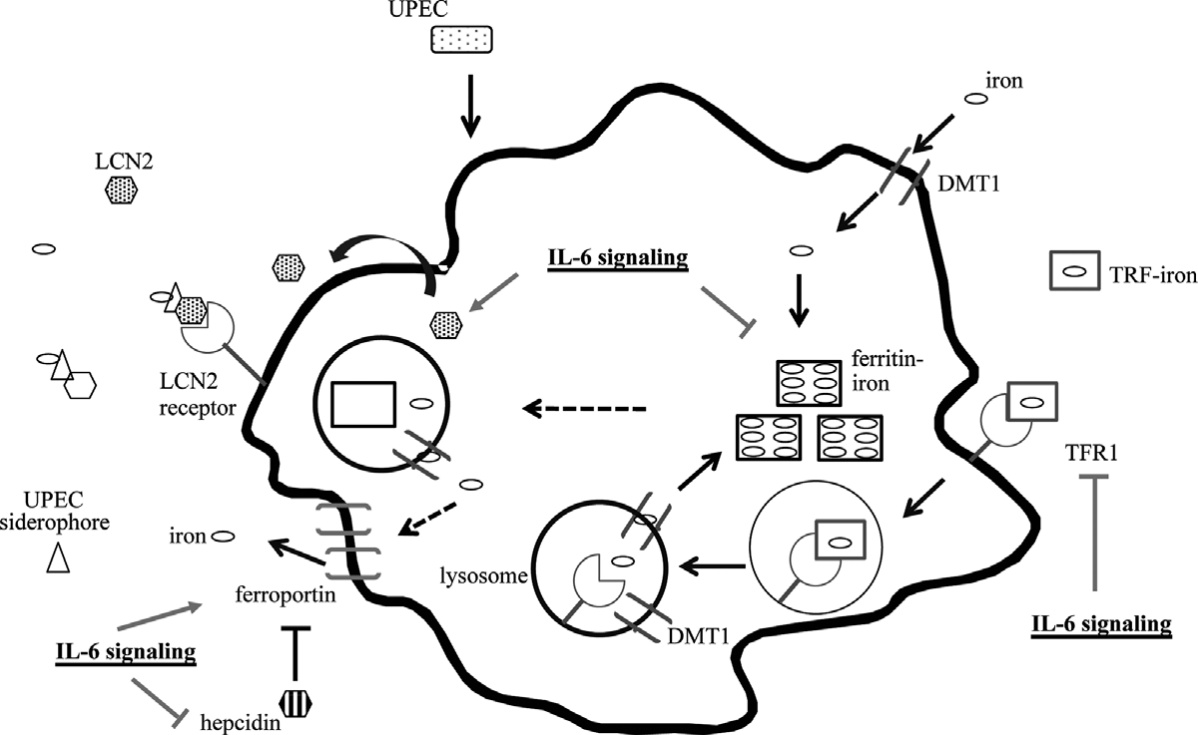
Model of macrophage iron retention during UPEC infection.

Here, we show that macrophages normally limit UPEC survival by restricting UPEC access to intra‐ and extra‐macrophagic iron stores. We also show that IL‐6 signaling is necessary for UPEC‐induced macrophage uptake of extracellular iron through lipocalin 2 production, while IL‐6 signaling negatively regulates UPEC‐induced macrophage iron retention. Furthermore, IL‐6 signaling is necessary for macrophages to limit UPEC survival under iron rich conditions. We propose a model wherein macrophages retain iron in response to UPEC, thereby keeping iron away from UPEC (see model in Fig. 6). Macrophages retain free iron by upregulating expression of *Dmt1*, an importer of extracellular free iron, thus allowing macrophages to take up free iron from the extracellular milieu and store the iron in a ferritin‐bound state. Macrophages, in response to UPEC, also negatively regulate expression of the iron exporter ferroportin and promote expression of hepcidin, a negative regulator of ferroportin, further limiting the ability of UPEC to access extracellular free iron. Furthermore, our data show that in response to UPEC, macrophages reduce expression of TFR1, a receptor that facilitates the release of iron from the extracellular cargo molecule transferrin. Reducing macrophage TFR1 expression thus furthers limit bacterial access to extracellular free iron.

Our findings are consistent with previous data showing that *C. pneumoniae* infection reduces macrophage expression of TFR1 42, while macrophages also increase *Dmt1* expression in response to other pathogens, such as *F. tularensis* 43. However, *F. tularensis* drives macrophage iron uptake in order to access iron within the macrophage and promote *F. tularensis* intra‐macrophagic survival 43. UPEC also exploits a number of mechanisms in order to persist within macrophages 30, 31. This contrasts with our model, in which macrophages limit the ability of UPEC to access iron both inside and outside of the macrophage. In further support of our model, we show that macrophages limit the ability of UPEC to use iron to promote their survival within macrophages.

A number of other mechanisms are known to govern macrophage iron regulation. Previous data suggest that the inflammatory cytokine IL‐6 regulates host iron retention 23 and the host‐response to UPEC 20, 21. Our data show that IL‐6 signaling did not significantly affect ferroportin‐mediated iron release by macrophages pre‐exposed to homeostatic iron levels. This is consistent with previous work showing that, under homeostatic iron conditions, IL‐6 deficiency did not significantly affect alveolar macrophage expression of hepcidin and ferroportin in response to lipopolysaccharide 44. However, our data suggests that IL‐6 signaling inhibits ferritin‐mediated iron retention and promotes ferroportin‐mediated iron release in macrophages pre‐exposed to high iron levels. This did not promote UPEC survival, since IL‐6 signaling inhibited UPEC survival within macrophages pre‐exposed to high iron levels. IL‐6 signaling instead limited UPEC access to extracellular iron by promoting lipocalin 2 production by iron‐supplemented macrophages, in agreement with previous data showing that lipocalin 2 reduces survival of *M. tuberculosis* and *C. pneumoniae* within macrophages 42, 45.

Macrophages pre‐exposed to homeostatic iron levels or high iron levels, produced lipocalin 2 and retained free iron in response to UPEC. This indicated that macrophages could perform their anti‐microbial iron regulation functions under a range of iron levels. This is especially relevant in vivo, since macrophages may be exposed to different concentrations of iron over the course of an infection 4. Interestingly, the host epithelial–iron interactions have the opposite effect on bacterial survival, as our previous work has shown that UPEC is able to access iron within bladder epithelial cells via ferritinophagy, thereby promoting UPEC survival and growth within these cells 19. We suggest that in the bladder mucosa, UPEC survival and growth within the bladder epithelium may be mitigated by the bladder macrophagic anti‐microbial iron regulation response, consistent with previous data showing increased bladder macrophage counts up to 48 hpi with UPEC 40. Our data strongly suggest that macrophages limit the ability of UPEC to access iron in vitro and that bladder macrophages produce key iron regulators such as lipocalin 2 in vivo upon UPEC infection. In line with this, previous studies have shown that loss of lipocalin 2 and hepcidin resulted in increased rates of pyelonephritis in mice and women 46, 47, 48. Further work is required to show that bladder macrophages guard against UTIsby limiting the ability of UPEC to access host iron.

Host iron homeostasis is known to be regulated by IL‐6, which induces hepcidin production by liver hepatocytes 22, 23, 24. In response to UPEC infection, both macrophages and bladder epithelial cells produce IL‐6 20, 21. Bladder macrophages are thought to prevent sub‐clinical UPEC colonization from transitioning into a pathological UTI 30, 36, 37. We therefore posit that macrophage IL‐6 signaling might play a crucial role in the macrophage anti‐microbial iron regulation response in infected tissues, since IL‐6 signaling both limited UPEC survival within macrophages pre‐exposed to high levels of iron and promoted lipocalin 2 expression by these macrophages. Bladder IL‐6 levels may regulate liver hepcidin production and subsequently alter the resident bladder macrophage response, thus allowing for increased iron sequestration during a UTI and limiting ferroportin expression in an autocrine fashion.

To overcome IL‐6‐induced lipocalin 2 defense, UPEC may elaborate lipocalin‐2‐resistant siderophores such as salmochelin 47, and inhibit IL‐6 production 49, 50 in order to limit lipocalin 2 production. In addition to promoting lipocalin 2 expression, IL‐6 signaling inhibited TFR1 expression by macrophages pre‐exposed to high iron levels, thereby limiting the liberation of free iron from transferrin. This may provide another explanation of why UPEC limits innate immune cell production of IL‐6: limiting IL‐6 production provides UPEC with access to free iron released from transferrin. Given this, activators of IL‐6 signaling could be a useful tool for modulating iron‐dependent host immune responses during UPEC infection. Together, our data provide new insights into how the innate immune system limits UPEC survival by limiting the ability of UPEC to access host iron. We propose that modulating host iron regulation response may provide an effective treatment for treatment of the UTIs caused by UPEC.

## Materials and Methods

### Mice

All protocols were approved by the animal studies committee of the Washington University School of Medicine (Animal Welfare Assurance #A‐3381‐01). Seven to nine‐week‐old C57BL/6 mice were used for studies involving wild type mice, and 7 to 9‐week‐old IL‐6^tm1Kopf^/J (the Jackson Laboratory) were used for IL‐6 knockout experiments.

### Isolation of murine peritoneal macrophages (pMacs) and bone marrow derived macrophages (BMDMs)

Bone marrow derived macrophages from wild type mice were isolated using previously published protocols 51. For pMac isolation, a 3.85% of thioglycollate (Difco, BD Bioscience Diagnostics) was made according to the manufacturer's protocol. Mice were injected intraperitoneally with 1 mL of 3.85% thioglycollate and allowed to rest for four days. Mice were sacrificed, and 3 mL of culture media (DMEM containing 10% FBS, 1% Glutamax, 1% penicillin/streptomycin; all from Gibco, Life Technologies), and 1% sodium pyruvate (Corning) was injected into the mouse peritoneal cavity. pMacs were then isolated using previously published protocols 25, 26, 52. Cells were centrifuged at 460×*g* for 5 min at 4**°**C, resuspended in culture media, counted, and plated at a density of 5 × 10^5^ cells/mL.

### Bacteria

Luria Broth media (Sigma) was inoculated with the UPEC clinical cystitis isolate UTI89 and grown as previously described 51.

### Cell culture

pMacs in a 24‐well tissue culture plate (∼5 × 10^5^ cells/well, 1 mL culture media/well) were treated for 24 h with “infection medium,” which was culture medium lacking penicillin/streptomycin, with or without 250 μM ferric ammonium citrate (66 μg/mL, FAC, Sigma–Aldrich, CAS: 1185‐57‐5) and with or without 125 ng/mL anti‐IL‐6Rα (R&D Systems, AF1830) or normal goat IgG isotype control antibody (R&D Systems, AB‐108‐C). Cells were infected with UTI89 in 1 mL of new infection media.

### Quantitative PCR (qPCR)

Following gentamicin treatment, infected pMacs were removed from the tissue culture plate using CellStripper (Corning), followed by trypsin treatment (Gibco, Life Technologies). The pMacs were then washed in 5% FBS, transferred to buffer RLT with β‐mercaptoethanol (Sigma, M6250), and frozen at −80**°**C, per manufacturer's protocols (RNeasy Mini kit, Qiagen). The samples were then thawed and RNA was isolated from the cells per manufacturer's protocols (RNeasy Mini kit, Qiagen). cDNA was then generated according the manufacturer's protocol, (First‐Strand cDNA synthesis Using SuperScript II RT, Life Technologies; with DNase treatment). qPCR was performed per manufacturer's protocols (SsoAdvanced Universal SYBR Green Supermix, BIO‐RAD, 1725274) using the following primers, with *Rn18s* serving as the control: *Hamp1* (hepcidin): 5′‐GCAGAAGAGAAGGAAGAGAGACACC‐3′ and 5′‐TGTAGAGAGGTCAGGATGTGGCTC‐3′;

*Fpn1* (ferroportin): 5′‐TGGAACTCTATGGAAACAGCCT −3′ and 5′‐TGGCATTCTTATCCACCCAGT‐3′; *Slc11a2* (*Dmt1*): 5′‐AACCAACAAGCAGGTGGTTGA‐3′ and 5′‐CCTTGTAGATGTCCACAGCCAGAGT‐3′; *Tfr1*: 5′‐GTGGAGTATCACTTCCTGTCGC‐3′ and 5′‐CCCCAGAAGATATGTCGGAAAGG‐3′; *Lcn2*: 5′‐TGGCCCTGAGTGTCATGTG‐3′ and 5′‐CTCTTGTAGCTCATAGATGGTGC‐3′; *Il‐6*: 5′‐CCAGAAACCGCTATGAAGTTCCT‐3′ and 5′‐CACCAGCATCAGTCCCAAGA‐3′; *Rn18s* (18S): 5′‐CGGCTACCACATCCAAGGAA‐3′ and 5′‐GCTGGAATTACCGCGGCT‐3′.

### Intracellular CFU analysis

pMacs were infected with UTI89 for 2 h in infection media and then washed in PBS. This was followed by either 100 μg/mL gentamicin for 1 h, or 100 μg/mL gentamicin for 1 h followed by 10 μg/mL gentamicin for 3 h. The pMacs were then washed in PBS. UPEC‐infected pMacs were then lysed in 1 mL of 0.1% Triton X‐100 (Ricca Chemical Company) for 10 min with moderate shaking. The bottom of the plate was then vigorously scraped with a 1 mL micropipette tip, and 5 μL samples of serially diluted lysate were plated on LB‐agarose plates. The plates were incubated 37°C overnight and colonies were counted the next day.

### Extracellular CFU analysis

pMacs were infected for either 3 or 6 h with UTI89 in 1 mL of infection media. A total of 5 μL of serially diluted media from these infected pMacs was then plated on LB‐agarose plates. The plates were incubated 37°C overnight, and colonies were counted the next day.

### ELISA

Supernatants were harvested from macrophage cultures after the indicated stimulations. IL‐6 was quantified using a DuoSet IL‐6 ELISA kit (R&D Systems) per the manufacturer's instructions.

### Western blots

pMac cell lysates were electrophoresed on 4–20% Pierce Precise Protein Gels (Bio‐Rad) and transferred to PVDF membranes. The membranes were treated with the following antibodies at the following dilutions: FTH1 (ferritin) rabbit polyclonal (1:1000; Cell Signaling, D1D4), total STAT3 (1:250; Cell Signaling, D3Z2G), pSTAT3 Tyr705 (1:100; Cell Signaling, D3A7), IL‐6 (1:100; Cell Signaling, D5W4V, Mouse Specific), and beta‐actin (1:500; Cell Signaling, 8H10D10).

### In vivo infection

Mice were briefly anesthetized with isoflurane. The mice were then transurethrally injected with 50 µL of PBS or 50 µL of UTI89 by using a 1 mL syringe and a catheter with a 26G needle. After 6, 24, or 72 h, mice were euthanized by exposure to isoflurane followed by cervical dislocation.

### Isolation of murine bladder macrophages

Bladders from euthanized mice were removed and briefly washed in PBS. Individual bladders were then placed into 1 mg/mL collagenase (Sigma) and 22 µg/mL DNAse (Sigma) in PBS or RPMI at 37**°**C. The bladders were subsequently shaken at 37**°**C for 30 min to 1 h, processed into a single‐cell suspension by using a 1 mL syringe plunger, and passed through a 70 µm filter. Bladder cells from the same treatment group were then pooled.

### Flow cytometry

Bladder macrophages were treated with TruStain fcX (anti‐mouse CD16/32; BioLegend) in 5% FBS (Gibco, Life Technologies) for 20 min. Cells were then washed, treated with the appropriate fluorescent antibodies and isotype control antibodies at the noted concentrations (see below) at 4**°**C for 20 min, and then washed with 5% FBS. Samples containing a biotin‐conjugated antibody were subsequently treated with the appropriate streptavidin‐conjugate at 4**°**C for 20 min. Cells were then washed and analyzed on a BD LSR II flow cytometer. The following antibodies from eBioscience were used at 1:50: CD11b‐APC (17‐0112‐83) and F4/80‐PE (12‐4801‐82). The following antibodies from BioLegend were used at 1:50: CD11b‐biotin (101204), CD64‐PE‐Cy7 (139314), and CD45‐Per‐CP‐Cy5 (103132). The following streptavidin conjugates from Life Technologies were used at 1:15: Strepatividin‐Alexa405 and Streptavidin‐PacificOrange.

### Cell sorting

A single‐cell suspension of bladder macrophages was stained per the above flow cytometry procedure, except 5% FBS with 25 mM HEPES substituted for 5% FBS. Cells were then isolated using a BD FACS Aria flow cytometer.

### Microarray analysis

Sorted bladder macrophages were transferred to buffer RLT with β‐mercaptoethanol (Sigma, M6250), with at least 6 × 10^3^ cells being per sorted bladder cell population. RNA was then isolated from the cells and frozen, per the manufacturer's protocols (RNeasy Mini kit, Qiagen), and assessed for quality using the Bioanalyzer 2100 (Agilent). With the Sigma WTA2 RNA amplification kit, 1.0 ng of total RNA were amplified and then 3 µg of cDNA were chemically labeled with Kreatech ULS Fluorescent Labeling Kit for Agilent arrays (Kreatech Diagnostics). Finally, 1.7 µg of labeled cDNAs were subsequently hybridized onto Agilent Mouse Gene Expression 4 × 44 K Microarrays (cat. G4122F‐014868). Slides were scanned on an Agilent C‐class Microarray scanner to detect Cy5 fluorescence, according to manufacturer's specifications. Gridding and analysis of images was performed using Feature Extraction (v11.5.1.1, Agilent Technologies). The data was then analyzed, and normalized heat maps were generated, using the R software package, with a cutoff of *P* ≤ 0.05 and fold change of >2.

### Statistics

Statistical analysis was performed using GraphPad Prism software. All results are expressed as means ± S.E. Groups were compared by paired Student's *t* test or two‐way analysis of variance as appropriate. A value of *P* ≤ 0.05 was considered significant.

## Conflict of Interest

None declared.
